# Estimating the Potential Public Health Impact of Prohibiting Characterizing Flavors in Cigars throughout the US

**DOI:** 10.3390/ijerph16183234

**Published:** 2019-09-04

**Authors:** Brian L. Rostron, Catherine G. Corey, Enver Holder-Hayes, Bridget K. Ambrose

**Affiliations:** Center for Tobacco Products, US Food and Drug Administration, Silver Spring, MD 20993, USA

**Keywords:** cigars, flavors, tobacco, mortality, initiation

## Abstract

Flavored cigar use is common among cigar smokers, particularly those at younger ages. Several US localities have implemented policies restricting the sale of flavored tobacco products, including cigars. We estimated the population health benefits of removal of flavored cigars throughout the US in terms of reductions in cigar smoking-attributable mortality due to increased cessation and reductions in cigar smoking prevalence due to decreased initiation and continuing use. Monte Carlo simulation was used to estimate possible ranges for these values. We used published estimates of cigar use and attributable mortality in the US, as well as prior study conclusions on the effect of local and national flavor restriction policies. We estimated that removal of flavored cigars would result in approximately 800 (90% prediction interval = 400–1200) fewer cigar smoking-attributable deaths in the US each year and 112,000 fewer cigar smokers (90% prediction interval = 76,000–139,000) in each cohort of 18 year olds. The removal of characterizing flavors in cigars sold in the US is thus projected to have substantial public health benefits over time.

## 1. Introduction

Flavored cigar use is a topic of considerable public health concern in the US. According to the 2016 National Survey on Drug Use and Health (NSDUH), over 12 million Americans smoked cigars in the past month [1]. Cigar use causes numerous serious health conditions, including heart disease and lung cancer [2] and it has been estimated that regular cigar use causes approximately 9000 premature deaths in the US each year [3]. Flavorings are added to tobacco products such as cigars to increase appeal in a variety of ways, such as improving the taste and sensory perceptions of the products, and by reducing harshness and bitterness associated with use [4]. The market share of flavored products in several tobacco categories, including cigars, has grown in the US in recent years [5,6]. According to an analysis of convenience store data, sales of flavored cigars increased by almost 50% from 2008 to 2015 and now represent more than half of the sales volume of cigars sold in these establishments [7].

Flavored cigar use is very common among cigar smokers, particularly at younger ages. Among youth aged 12–17 years in the Population Assessment of Tobacco and Health (PATH) Study in 2013–2014, 65% of ever cigar users reported that their first cigar was flavored and 72% of past 30 day users reported flavored cigar use [8]. These results are consistent with estimates from the 2014 National Youth Tobacco Survey (NYTS), which found that 64% of middle and high school cigar smokers used flavored cigars, representing a total of 910,000 youth. Young cigar users also consistently report that flavors are a reason for use. For example, 73% of youth in Wave 1 of the PATH Study from 2013–2014 who had used cigars in the past month endorsed appealing flavors as a reason for using cigars [9]. Patterns and reasons for use at older ages share some similar characteristics. Data from the National Adult Tobacco Survey (NATS) from 2013–2014 showed that 36% of adult cigar users, or 4.1 million individuals, had used flavored cigars in the past month and among young adults aged 18 to 24 years, the figure was 48% [10]. Among PATH young adult cigar smokers, 82% cited flavors as a reason for use, with the figure being 67% for cigar smokers aged 25 years and older [9]. Wave 1 PATH data also showed that more than half of adult established filtered cigar, cigarillo, and non-premium traditional cigar users reported that their favorite or most recent cigar brand was flavored [11].

Because of these issues, several US localities, including New York City, Minneapolis, St. Paul, Minnesota, and Providence, Rhode Island, as well as the nation of Canada, have implemented policies restricting the sale of flavored tobacco products, including cigars [12,13,14,15]. These policies have been found to be associated with decreased tobacco use among young people [13], as well as decreased total consumption of tobacco products, such as cigars [12]. The implementation of similar policies throughout the US could reduce initiation and use of cigars among youth and young adults while encouraging cessation among existing adult cigar smokers. This analysis quantifies the potential public health benefits of extension of existing policies prohibiting the sale of cigars with characterizing flavors, other than tobacco, to states and localities throughout the entire US. Benefits are estimated both in terms of the number of young people deterred from cigar initiation and use, and the number of premature deaths avoided by increased cessation among current users. Because of the inherent uncertainty about the future behavioral and health effects of policies prohibiting characterizing flavors in cigars, the analysis also utilizes a simulation approach that allows for variability in key input values and projects a range of estimates for benefits.

## 2. Materials and Methods

### 2.1. Mortality Benefits from Increased Cessation

We estimated benefits among existing cigar users due to these extended policies in terms of reduced premature mortality caused by increased cigar smoking cessation. We began with published estimates of mortality due to regular cigar use in the US each year [3] and only considered deaths among exclusive cigar smokers. This was because users of additional tobacco products such as cigarettes may continue to use those other products after implementation of the policies, instead of quitting tobacco use entirely.

Next, we considered the possible effects of the policies on cigar consumption. We used observed data on overall cigar sales from two localities, New York City and Providence, Rhode Island, that have enacted policies limiting the sale of flavored tobacco products, including cigars. Rogers et al., [12] analyzed data on total cigar sales in New York City after the city restricted the sale of flavored tobacco products in 2010 and found that the policy was associated with a reduction in total cigar unit sales of approximately 15% to 20%, relative to surrounding areas and the US overall. They also noted that sales of flavored cigars continued at high levels in the city after the restriction, perhaps attenuating the policy’s effect on reducing cigar use. Rogers et al. [16] also analyzed similar data from Rhode Island after implementation of a flavored product restriction in Providence in 2013 and found that the policy was associated with a 31% reduction in total cigar sales in the city. We therefore used the figure of 30% as our main estimate for reduced cigar consumption due to the policies. We set 15% as the lower estimate, based on the reduction in total cigar sales in New York City even with incomplete compliance. Euromonitor data [17] show that 92% of cigars sold in the US are machine-made, and Delnevo et al. [7] found that flavored cigars constituted 52.1% of the market share of cigars sold in convenience stores in the US in 2015. Assuming that the share of machine-made cigars sold through other retail channels is similar and that hand-made cigars are generally not flavored, then the proportion of cigars sold in the US that are flavored is approximately 45% (~ 92% * 52.1%). We used this figure as the upper estimate for the reduction in cigar consumption in our analysis.

We then estimated the proportion of the reduction in cigar use that would be caused by complete cigar smoking cessation, as opposed to reductions in cigar consumption. We used data from the International Agency on Research on Cancer [18], which has found that studies of the effects of tax increases on cigarette sales suggest that approximately half of observed reductions in cigarette sales are due to smokers quitting entirely, with the remainder due to smokers cutting back on the number of cigarettes smoked. We assumed that complete cessation would reduce mortality risks for existing exclusive cigar smokers and did not assign any reduction in mortality risk to cigar smokers who reduced their cigar consumption but did not quit tobacco use entirely. Given that analysis of National Health Interview Survey (NHIS) data has shown that prevalence of cigar use among US adults remained generally stable from 2000 to 2015 [19], we assumed that cigar smoking prevalence and thus cigar smoking-attributable mortality in the absence of policies on flavors, would remain relatively constant over time.

Table 1 lists the data inputs and values that were used to quantify the mortality benefits of the extended policies from increased cessation among existing cigar users. As sensitivity analysis, we conducted a Monte Carlo simulation in @RISK modeling software [20] for 1000 iterations that sampled values from triangular probability distributions for key inputs. The minimum, peak, and maximum values for the reduction in cigar consumption were 15%, 30%, and 45%, and the values for the proportion of the reduction that would be due to complete cessation were 25%, 50%, and 75%.

### 2.2. Changes in Use Prevalence Due to Deterred Initiation and Continuing Use

We also estimated the number of young people who would be deterred from initiating or continuing cigar use due to the extended policies. We began with the cohort of 18 year olds in the US in 2016 and calculated the number who were current cigar smokers at that age, as well as the number of these current users who had initiated cigar use with flavored products, based on PATH data on cigar use among 18 year olds and cigar initiation among youth [8]. We then considered the proportion of these cigar users who would have initiated with non-flavored cigars in the absence of flavored cigars. We assumed that the lower estimate would be 35%, equal to the proportion of cigar users who currently initiate with non-flavored products, and that the upper estimate would be 100%, which assumes complete substitution with non-flavored cigars. We used the midpoint of these values, 67.5%, as our main estimate, so 32.5% of those currently initiating with flavored cigars would be deterred from trying cigars. We also accounted for the possibility that flavored cigar initiates are more likely to continue cigar use than those who initiate with non-flavored products, using PATH data that showed that adult ever cigar users who had initiated with flavored cigars were more likely to be current regular cigar users than ever users who initiated with non-flavored cigars (adjusted prevalence ratio = 1.56, 95% confidence interval = 1.29, 1.87), controlling for other relevant factors related to cigar use and progression [21].

**Figure 1:**
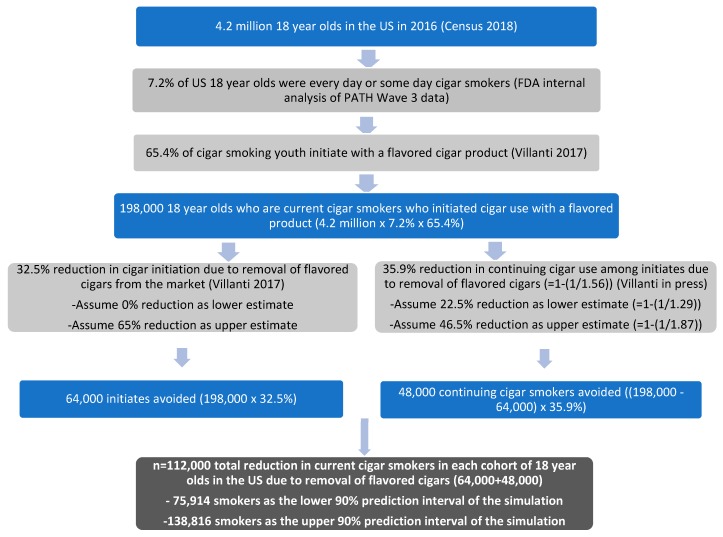
Calculations used to estimate the potential number of new cigar initiates and continuing cigar smokers that may be averted by policies prohibiting flavored cigars throughout the US.

Figure 1 shows the steps used to quantify the reduction in cigar initiation and use among young people that would be produced by the policies. We conducted a simulation for 1000 iterations in ^@^RISK with triangular probability distributions. The minimum, peak, and maximum values for the reduction in cigar initiation were 0, 32.5%, and 65%, and the values for the reduction in continuing use among those who would have otherwise initiated cigar use with flavored cigars were 22.5%, 35.9%, and 46.5%.

## 3. Results

For the mortality benefit from increased cessation among existing cigar users, we estimated that there would be a 15% reduction in mortality from cigar smoking, due to a 30% reduction in total cigar consumption of which 50% would come from complete cessation. We therefore calculated that, among the 5200 current premature deaths from exclusive regular cigar use [3], there would be 780 (5200 * 30% * 50%) premature deaths avoided each year because of the policies.

In the simulation of reduction in cigar consumption with values of 15%, 30%, and 45%, and the proportion of the reduction that would be due to complete cessation with values of 25%, 50%, and 75%, we obtained a mean estimate of 779 deaths avoided, with a 90% prediction interval of 440 to 1194 deaths avoided. We also examined the effects of particular input values on mortality estimates. Varying the reduction of cigar consumption across its range of values, while holding the proportion of reduced consumption that was due to complete cessation constant, resulted in 522 to 1057 deaths avoided. Similar variation of the proportion of reduced consumption that was due to complete cessation, while holding the reduction in cigar consumption constant, produced a range of 509 to 1055 deaths avoided.

For the reduction in cigar initiation and use, we began with the 4.2 million 18 year olds in the US in 2016 and estimated that 7.2% of them would be current cigar users at that age, based on an internal analysis of PATH Wave 3 data for self-reported every day or some-day cigar use. This figure may be somewhat conservative because 13.1% of 18 year olds in PATH reported cigar use in the past 30 days, which is the measure commonly used for tobacco use prevalence among youth. We also used PATH data [8] to estimate that 65.4% of these cigar smokers would have initiated cigar use with a flavored product. Among the resulting 198,000 18 year olds who are currently using cigars and had initiated cigar use with a flavored product, we estimated in the main analysis that there would be a 32.5% reduction in initiation of use for a total reduction of 64,000 (198,000 * 32.5%) initiates. We also estimated that there would be a 35.9% (1–(1/1.56)) reduction in continuing use among initiates due to the removal of flavored cigars from the market, which results in an estimate of 48,000 (198,000 * (1–32.5%) * 35.9%) fewer continuing cigar users because of the policies. The total reduction in current cigar smokers would therefore be 112,000, as shown in Figure 1. This estimate represents a reduction in current cigar prevalence of 37.1% for each cohort of 18 year olds.

In the simulation with reductions in cigar initiation with values of 0%, 32.5%, and 65%, and reductions in continuing use among those who would have otherwise initiated cigar use with flavored cigars with values of 22.5%, 35.9%, and 46.5%, we obtained a mean estimate of 108,846 current users prevented with a 90% prediction interval of 75,914 to 138,816 users prevented. Variation in the reduction in cigar initiation across its range of values, holding the reduction in continuing use constant, had a large effect on estimates and resulted in 78,298 to 138,455 current users prevented. Similar variation in reduction of continuing use produced 96,393 to 120,380 users prevented.

## 4. Discussion

The estimates presented in this study have demonstrated the substantial possible public health benefits of measures prohibiting the sale of cigars with characterizing flavors other than tobacco in states and localities throughout the US. These benefits would result from reduced mortality among current cigar smokers through increased cessation and from reduced cigar use among young people because of decreased initiation and progression to regular use due to decreased appeal of these products. We find that extending policies that have been implemented in certain US localities on a nationwide basis could result in the prevention of approximately 800 premature cigar-attributable deaths in the US each year with a 90% prediction interval of approximately 400 to 1200 premature deaths avoided. We also find that such policies could result in a reduction of approximately 112,000 current cigar smokers in each cohort of 18 year olds in the US, with a 90% prediction interval of 76,000 to 139,000 users prevented.

These projections cannot predict with certainty the population health effects of policies that have yet to be implemented, but they do present a range of plausible estimates. Data inputs come from the best available empirical evidence, and the consistency of results from localities such as New York City and Providence, Rhode Island supports the notion that policies restricting the availability of characterizing flavors in cigars have the effect of reducing cigar use. This reduction can occur among existing and new cigar users, providing immediate health benefits from increased cessation for people at middle and older ages and long-term benefits from reduced initiation and continuing use among young people. The use of Monte Carlo simulation techniques has allowed for the quantification of the uncertainty inherent in projections of the effect of potential policies. In some ways, our estimates are conservative, given, for example, that we assumed that half of reduced cigar consumption would come from cigar smokers cutting back on but not quitting cigar use entirely and assigned no health benefit to this reduction in use.

It is possible that the benefits of these policies could be reduced by other effects. The implementation of policies restricting the sale of cigars with characterizing flavors in particular US localities and in Canada has demonstrated that their effectiveness depends in part on how the policies are formulated as well as on how thoroughly and effectively they are implemented and enforced [12,15]. In general, these measures would be most effective if they were comprehensive in nature and prohibited the manufacture and sale of flavored cigars. They would also be most effective if they were consistently implemented throughout the US. Another effect that has been observed in localities implementing flavor restrictions has been decreases in sales of cigars with specific flavor descriptors, such as candy or fruit, coupled with increases in sales of cigars with non-specific or concept descriptors that may serve to suggest the presence of flavors [16]. Various criteria could be used to assess the presence of characterizing flavors in cigars, including: The use of flavorings as ingredients, the presence of flavor descriptors or representations whether specific or non-specific in labeling or packaging, or the sensory experience of a flavor, such as taste or aroma during product use.

The benefits of these measures could also be offset to some extent if cigar smokers were to use other combustible tobacco products such as cigarettes instead. To some extent, we address this issue in our projections by not assigning any benefits of the policies to current dual users of cigars and cigarettes. Although substitution with other tobacco products is a possibility, there is some evidence suggesting that total tobacco use among youth and young adults in Rhode Island has declined since the flavor ban was enacted in Providence [22]. The population health benefits of such policies could actually be greater than estimated in this analysis if flavored cigar use leads young people to then use other tobacco products such as cigarettes. There would also be health benefits from the policies, in addition to decreased premature mortality. For example, it has been estimated that exclusive former cigar smokers in the US have approximately 200,000 cases of cardiovascular disease and cancer that are attributable to cigar use [19]. Such morbidity and related disability would be decreased by increased cessation and decreased initiation of cigar use. Finally, this study only examined potential population health benefits of policies regarding flavored cigars, and there could be additional benefits from comprehensive policies that address flavored tobacco products generally.

This analysis is subject to certain limitations. We have used the best available data inputs that we are aware of, but information on the health effects of cigars and the effects of flavor bans for cigars is somewhat limited. For example, we based our mortality estimates on published estimates of mortality due to regular cigar use, defined as having used cigars on 15 of the past 30 days. There could be some additional mortality due to less frequent cigar use that was not captured in these previous estimates. In addition we used empirical evidence from localities such as New York City and Providence with existing bans to estimate the effect on cigar use, but it is not known whether results in these cities would be the same for the entire country. Many of the inputs came from data on use and health effects of cigars generally, given that previous studies and surveys often did not ask about particular cigar types. Finally, future cigar use and health effects will depend on consumer behavior, and regulatory and industry actions, which cannot be anticipated at this time.

## 5. Conclusions

Our projections provide further evidence that prohibiting cigars with characterizing flavors nationwide would benefit the US public health. We estimate that extending existing such policies would eliminate hundreds of premature deaths among current cigar smokers and prevent tens of thousands of young people from becoming regular cigar smokers in the first place each year.

## Figures and Tables

**Table 1:** Data inputs used to estimate the potential number of cigar attributable deaths prevented by policies prohibiting flavored cigars throughout the US.

Data Input	Value	Source
Premature deaths from exclusive regular cigar smoking in the US each year	5200 deaths	Nonnemaker et al. [3]
Reduction in cigar consumption due to flavor policies for cigars	30% in main analysis	Based on Providence, RI data in Rogers et al. [16]
	15% and 45% as lower and upper estimates	15% based on New York City data in Rogers et al. [12] 45% based on estimated flavored cigar market share (92% of cigars are machine-made (from Euromonitor data) and flavored cigars constituted 52.1% of convenience store cigar sales in 2015 [7])
Proportion of reduction in cigar consumption due to complete cessation	50% in main analysis	Data on effects of cigarette tax increases on reductions in cigarette sales due to smokers quitters entirely [19]
	25% and 75% as lower and upper estimates	---
Main Mortality Estimate	5200 * 30% * 50% = 780 premature deaths avoided each year	---
